# Evaluating the Impact of Age on Prostate Cancer Overdiagnosis Using Long‐Term Follow‐Up From the CAP Randomised Trial

**DOI:** 10.1002/ijc.70492

**Published:** 2026-04-22

**Authors:** Adam R. Brentnall, Matejka Rebolj, Peter Sasieni, Garth Funston, Rhian Gabe, Andrew J. Vickers

**Affiliations:** ^1^ Wolfson Institute of Population Health Queen Mary University of London London UK; ^2^ Memorial Sloan Kettering Cancer Center New York New York USA

**Keywords:** overdiagnosis, prostate cancer, PSA testing, screening

## Abstract

Prostate cancer overdiagnosis is detection of prostate cancer through PSA testing that otherwise would not have been diagnosed within the patient's lifetime. It is a major concern to policymakers due to its impact on quality of life. We used long‐term follow‐up data from the CAP randomised trial of a one‐off screen, and English male competing mortality rates (2021–23), to estimate the impact of age on excess prostate cancer incidence within 15 years (‘overdiagnosis’) using competing‐risks methods. In total, 2249 (1.19%) of 189,386 men invited for a PSA test in CAP had cancer detected at the one‐off screen. Prostate cancer cumulative incidence at 15 years was 7.08% (95% CI 6.95%–7.21%) in those invited to screening, compared with 6.94% (95% CI 6.82%–7.06%) in the control arm; an absolute excess incidence difference of 0.14% (95% CI −0.04% to 0.37%). Excess net incidence to 15 years was 0.14/1.19 = 11.7% (95% CI 0.0%–26.7%) of cases detected at a single prevalent screen. Accounting for competing mortality, men diagnosed at screening aged 50 years were projected to have a 16% chance the cancer would not have been detected within 15 years, rising to 32% if detected at screening aged 70 years and 58% aged 80 years. Thus, prostate cancer overdiagnosis rises substantially with age due to competing mortality, and is relatively low for younger men. Accordingly, policies that enable opportunistic testing should be re‐examined in settings where they have led to high rates of screening in older men.

## Abstract (highlights)

What's new?

Uncertainty persists over whether the benefits of prostate‐specific antigen (PSA) screening outweigh harms from overdiagnosis, a key barrier to population‐wide adoption. Here, English male mortality rates were analyzed alongside long‐term data from the UK Cluster Randomized Trial of PSA Testing for Prostate Cancer (CAP) to estimate age‐specific overdiagnosis. Excess prostate cancer detection was relatively low overall but increased sharply with age of screening detection, reaching 58 percent at age 80. The findings indicate that overdiagnosis is driven largely by competing mortality and is low in younger men, supporting more targeted, age‐informed screening policies and reconsideration of widespread testing in older populations.

## Graphical abstract

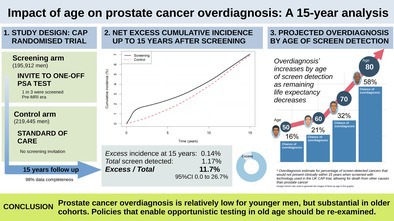

**Abbreviations**
CAP: UK Cluster Randomised Trial of Prostate Screening
CI: confidence interval
ERSPC: European Randomised study of Screening for Prostate Cancer
HR: hazard ratio
LUTS: lower urinary tract symptoms
MRI: magnetic resonance imaging
NSC: National Screening Committee
PSA: prostate specific antigen
SE: standard error
UK: United Kingdom

## 1 Introduction

The evidence that prostate‐specific antigen (PSA) screening reduces prostate cancer mortality has become clearer with extended follow‐up from two large randomised trials [1, 2]. However, there remains considerable uncertainty whether the benefits of PSA screening, in terms of reduced mortality, outweigh the harms from PSA testing, in particular overdiagnosis. Some guideline developers, including in the European Union, now recommend risk‐based PSA screening [3]. However, many high‐income countries, including the UK, have not recommended population‐based PSA screening, largely on the grounds of the harms estimated from overdiagnosis [4]. Prostate cancer overdiagnosis is detection of prostate cancer through PSA testing that otherwise would not have been diagnosed within the man's lifetime. It occurs when a man, or other person with a prostate, has prostate cancer detected from an opportunistic or organised programme screening, but dies from other causes before the cancer would have presented clinically following symptoms without screening. The main harms due to overdiagnosis are from subsequent disease management that negatively impacts quality of life, and related costs to the patient and healthcare system. Men with overdiagnosed prostate cancer have an increased risk of, among others, incontinence, erectile dysfunction, bowel irritation, and anxiety if the prostate cancer is monitored for signs of progression. The risk of harm in men screened has been reduced but not eliminated through the use of magnetic resonance imaging (MRI) in the diagnostic pathway [5]; approximately 8% of men diagnosed with low‐risk cancer in England and Wales nonetheless received radical treatment in 2021 [6].

Accurate evaluation of overdiagnosis is difficult. In theory overdiagnosis could be observed directly in a study where men had PSA tests and diagnostic workup but were blinded to whether they had prostate cancer detected. Men who subsequently died without a prostate cancer diagnosis over the rest of their life, but who had it detected at screening, would have been overdiagnosed by screening. Of course, such a study design would be unethical and impractical and so evaluation of overdiagnosis requires indirect methods. One method used is modelling, including microsimulation approaches where the natural history of prostate cancer is simulated in a cohort of digital humans. This approach was done to inform prostate cancer screening recommendations by the UK National Screening Committee [7]. A difficulty of such models is that they require many assumptions. Any approach that depends on a large number of assumptions, many of which cannot be tested, needs to be shown to be well calibrated to empirical data before projections are accepted. For instance, when the model is used to compare a new policy with current standard of care, the model should match epidemiological parameters such as incidence and mortality. Since overdiagnosis is a key aspect of the harm/benefit equation, we also believe that model‐based estimates of overdiagnosis should also have face validity against empirical estimates of the excess of cancers that would be diagnosed over a person's lifetime as a result of screening, versus no screening. This motivated us to conduct a re‐analysis of data from a mature randomised trial with a stop‐screen design and long follow‐up after screening [1]. Some have considered this to be, in general, the gold standard empirical approach to estimate overdiagnosis [8], subject to having (often difficult to achieve) suitable conditions for unbiased estimation that include stable testing patterns after screening ends with follow‐up that approaches the maximum preclinical period [9, 10]. A particular challenge for estimation of excess prostate cancer incidence is the need for follow‐up potentially several decades beyond the last screen, due to the known long lead time of PSA testing. In this paper we contribute a novel analysis that extends the concept of overdiagnosis to consider excess diagnoses as a function of time since last screen, and age at screening. We believe that such an analysis on excess incidence from a randomised controlled trial provides high quality causal evidence on the extent to which men with screen‐detected prostate cancer would have also been diagnosed later in life without screening, and a robust means to evaluate validity of model‐based estimates of overdiagnosis, conditional on length of follow‐up. It is also unlikely that we will have better data to estimate overdiagnosis with modern diagnostic pathways using the excess incidence approach from other studies anytime soon. Therefore, we need to make the best use of such data to inform policy now.

## 2 Materials and Methods

The UK Cluster Randomised Trial of PSA Testing for Prostate Cancer (CAP) trial evaluated a one‐off prostate cancer PSA test [1]. Between 2001 and 2007, *n* = 195,912 men were included in general practices (GPs) that were cluster randomised to a one‐off invitation for a PSA screening test, and *n* = 219,445 men in control GPs. A screen‐positive test result was indicated if PSA was above 3.0 ng/mL. Ten‐core transrectal ultrasonography‐guided biopsies were used for further investigations. Follow‐up data on vital status and cancer registration through December 21, 2020 were from National Health Service databases. Follow‐up data were complete for 97% randomised to the intervention and 99% to control, up to December 31, 2020, with a median of 15 years (IQR: 14.2–16.4; range, 12.2–19.2) after randomisation. The trial took place in a setting and era when there was relatively little opportunistic PSA testing in the target population. *n* = 64,425 (33%) men randomised to screening had a valid test result, and cumulative PSA testing in the control arm was estimated by the trialists to be 10%–15% over the first 10 years [1].

We evaluated one of the epidemiological definitions of overdiagnosis using excess cumulative incidence $O\left(t\right)$ to t years after a one‐off screen, considering the absolute difference in risk $P_{A}\left(t\right)-P_{B}\left(t\right)$ between those randomised to screening arm A versus control arm B, relative to the absolute risk of screen‐detected cancers at the one‐off screen $R_{A}$:

$$O\left(t\right)=\frac{P_{A}\left(t\right)-P_{B}\left(t\right)}{R_{A}}.$$

Using $R_{A}$ as the denominator rather than, for example, $P_{B}\left(t\right)$, has an advantage that the measure is less affected by the degree of compliance to the intervention in the trial. Excess incidence as a measure of overdiagnosis also avoids the limitation of a morphologic definition of overdiagnosis as limited to detection of indolent cases. However, interpretation of excess incidence measures for lifetime overdiagnosis requires a long follow‐up time t=T [11]. The median 15‐year follow‐up since screening in CAP is substantially longer than the time since last screening in other prostate cancer screening trials with (repeat) PSA testing [1, 2, 12]. For shorter follow‐up, the formula for $O\left(t\right)$ does not measure overdiagnosis but observed cumulative excess to a given time t. This need not correspond to overdiagnosis if the expected ‘compensatory drop’ in incidence in the screening arm has not ended, so that the excess could decrease further beyond the latest follow‐up. The compensatory drop in incidence is expected once screening stops, because some cancers that would have been diagnosed then were diagnosed early from screening.

With $n_{A}:n_{B}$ randomisation and complete follow‐up to T years after screening (no censoring other than death), overdiagnosis may be estimated using the observed cumulative number of cancers $Y_{j}\left(T\right)$ through $P_{j}\left(T\right)=Y_{j}\left(T\right)/n_{j}$ for j=A,B, with $R_{A}$ as the percentage of those randomised with screen‐detected cancer at the one‐off screen. An issue to contend with is that subjects are sometimes are censored before time T in a trial. This might occur for several reasons, including randomisation taking place over an extended period of calendar time, loss to follow‐up, or death. If the censoring process is independent of prostate cancer incidence, then one may estimate net risk where the impact of the censoring process is removed in the standard way, by using the complement F=1−S of Kaplan–Meier estimates. This estimates the incidence of prostate cancer irrespective of censoring, and so in the CAP trial data, irrespective of death from other causes. One may then define ‘net’ excess $E\left(t\right)$ at t years after screening using:

$$\begin{array}{cc} E\left(t\right)=\frac{F_{A}\left(t\right)-F_{B}\left(t\right)}{R_{A}}. & \end{array}$$

**FIGURE 1:**
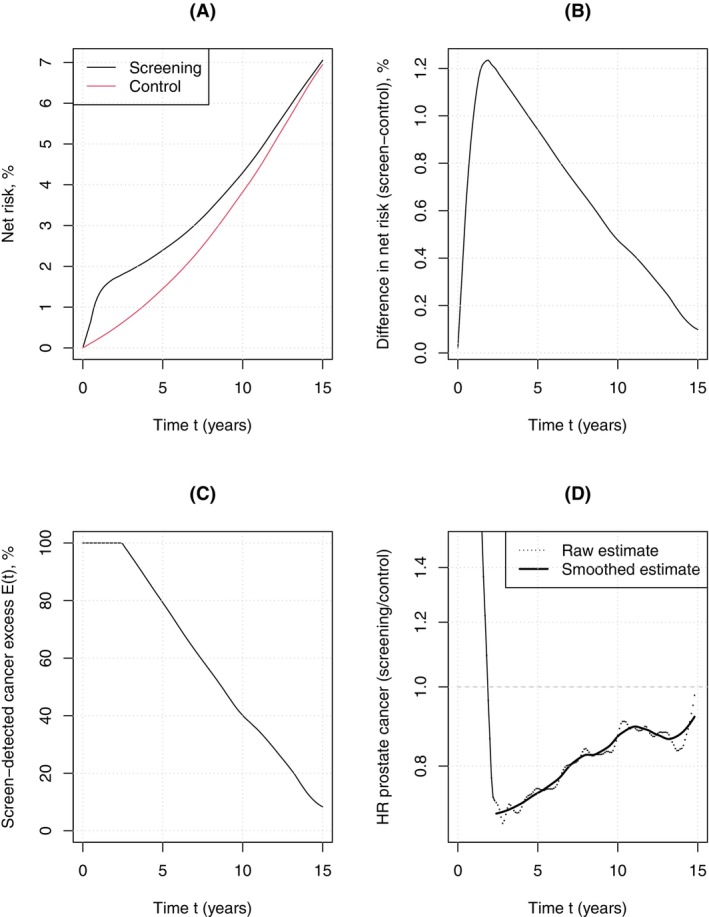
Estimates of net overdiagnosis and excess cancer incidence using data reported 0 to 15 years follow‐up from the CAP trial. Panel (A) shows the net risk estimates (Kaplan–Meier) [1], (B) is the absolute difference in net risk between the screening and control arm from panel (A); (C) is the observed excess $E\left(t\right)$ as a function of follow‐up time t, being the *y*‐value from plot (B) divided by a baseline screen detection rate of (2249/189,384) [13], capped at 100% during early follow‐up, leading to an estimate of overdiagnosis at t=15 years; (D) shows the ‘compensatory drop’ of cancer incidence following screening using an estimate of the hazard ratio (HR) for prostate cancer incidence as a function of follow‐up time, based on data from (A). [Color figure can be viewed at wileyonlinelibrary.com]

To estimate net excess from CAP we extracted aggregate Kaplan–Meier estimates of prostate cancer incidence by arm and follow‐up time 0<t≤15 (figure 2c of [1] using https://plotdigitizer.com/; our Figure 1A). Then to ensure overdiagnosis was not greater than 100%, we estimated the net excess at t as $E\left(t\right)=\min\left[1,\left\{F_{A}\left(t\right)-F_{B}\left(t\right)\right\}/R_{A}\right]$. We set $R_{A}=1.19\%$ because it has been reported that 2249 (1.19%) of 189,386 men analysed in the PSA arm in CAP had cancer detected at the baseline screen [13]. To estimate overdiagnosis it is necessary to consider two competing risks: (1) net excess, and (2) competing mortality. For example, if everyone died within 1 year (e.g., due to a natural disaster) then overdiagnosis would be 100%. If nobody died within 15 years (~impossible) then net excess would equal crude excess. The truth will lie in between these extremes depending on rates of competing mortality, with overdiagnosis being higher for those with higher competing mortality rates such as older men. To proceed we assumed that net excess is independent of competing mortality. This is reasonable for prostate cancer, as it is not strongly linked with other causes of death. We further assumed net excess is independent from age; a weaker assumption than assuming that incidence is independent from age (see discussion for evaluation of risk of bias from this assumption). Then we applied standard competing‐risks methodology, treating $E\left(t\right)=S_{1}\left(t\right)$ as a survival function (for the first competing risk) [14, 15]. For the second competing risk, competing‐mortality rates $h_{2}\left(a\right)$ by year of age a were taken from all‐cause English male mortality rates 2021–23 [16], subtracting age‐specific prostate‐cancer mortality rates [17]. Annual excess rates were calculated as $h_{1}\left(k\right)=\log\left\{S_{1}\left(k-1\right)\right\}-\log\left\{S_{1}\left(k\right)\right\}$ by year since randomisation k=1,2,…,15. Then crude excess for a man aged a years, t years after screening, was from:

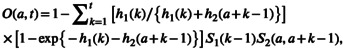

where $S_{2}\left(b,c\right)=1-\exp\left\{-\sum_{a=b}^{c-1}h_{2}\left(a\right)\right\}$ for c>b; $S_{2}(b,b)=1$. Overdiagnosis by age a was projected excess at 15 years, $O\left(a,15\right)$. Further analysis on the closing of the compensatory drop evaluated the hazard ratio for incidence from the trial as a function of follow‐up time using the gradient of the derived cumulative hazard function, and a smoother. Confidence intervals from reported summary statistics were estimated using a *z*‐statistic with standard errors (SEs) from reported confidence intervals (95% confidence interval width divided by 2 * 1.96). All analysis was done in the statistical software R, and source code and data used are fully available.

## 3 Results

### 3.1 The Impact of Follow‐Up Time on Excess Incidence

Prostate cancer net cumulative incidence was 7.08% (95% CI 6.95%–7.21%) at 15 years in CAP in those invited to a one‐off screen, compared with 6.94% (95% CI 6.82%–7.06%) in control; a net excess incidence difference of 0.14% (95% CI −0.04% to 0.37%; Figure 1B) [1]. Equivalently, relative net excess incidence of prostate cancer at 15 years was approximately (a statistically non‐significant) 2% (7.06/6.94) higher. Since 1.19% of the screening arm were reported to have prostate cancer detected at the baseline screen [13], net excess to 15 years was 0.14/1.19 = 11.7% (95% CI 0.0%–26.7%) of cases detected at the single trial screen. The confidence interval is relatively narrow, being based on > 24 thousand men with prostate cancer, and it includes 0%. The role of follow‐up time on net excess incidence is illustrated in Figure 1C. Here the net excess with 5 years of follow‐up was approximately 80% of screen‐detected cases, so that at most approximately 20% of the cancers diagnosed at screening would have presented clinically in the first 5 years. This drops to 40% with 10 years follow‐up. Even with a median 15 years follow‐up after screening data from CAP do not appear fully mature, and it remains to be seen if the compensatory drop in cancer incidence has ended (Figure 1D), that is, whether PSA testing can identify cancers with a lead time of more than 15 years.

### 3.2 The Impact of Age on Overdiagnosis

The average English man aged 50 years has a 10% chance of death in the next 15 years, rising to 20%, 30%, 49% and 89% if they are respectively 59, 64, 70 or 80 years of age [16]. The ability of PSA testing to identify prostate cancer a decade or more early means that many more will be overdiagnosed due to death from other causes than prostate cancer, following PSA tests done at an older age. This can be quantified using Figure 1C, which suggests that the excess decreases from year 3 after screening by 7.36% per year {(100 − 11.7)/12} through to year 15. We used this assumption together with the competing mortality assumptions to estimate the proportion of screen‐detected cancers that would have been diagnosed within 15 years by age, allowing for different rates of death from other causes. These calculations projected that the average man alive in England aged 50 years and diagnosed with prostate cancer at screening has a 16% chance to not have been diagnosed with prostate cancer within 15 years if he had not attended screening. If the maximum sojourn time is 15 years (which would coincide with closing of the compensatory drop), then this is an estimate of overdiagnosis (but if the maximum sojourn time is more than 15 years then this estimate of overdiagnosis will be too high). To illustrate the impact of age further, for men aged respectively 59, 64, 70 and 80 years of age at PSA screen detection, overdiagnosis defined in this manner is estimated to be respectively 20%, 24%, 32% and 58%.

## 4 Discussion

### 4.1 The Main Driver for Overdiagnosis From PSA Testing Is Age

We evaluated overdiagnosis from a one‐off screen by using a competing‐risks analysis. For this we first estimated the net excess incidence from a large unique randomised trial with a one‐off (stop) screen design and long, complete registry follow‐up on the majority of participants (limiting risk of bias from informative censoring). To estimate overdiagnosis we assumed that net excess was constant by age and combined the estimate with national data on competing mortality rates in English men. The data from CAP showed that net excess incidence in trial participants from screening continues to decrease through to 15 years after screen detection and it is empirically unclear if the trend has ended at 15 years. This long lead time means that overdiagnosis increases substantially with age of screen detection due to competing mortality. While the importance of age is consistent with the broader literature [18, 19], our overdiagnosis estimates are lower for younger men than some other studies using the excess incidence method. The main reason is that analysis considered a long 15‐year follow‐up after screening. ERSPC is the only other large randomised trial with complete recruitment that may generate suitable data to estimate overdiagnosis via the excess‐incidence method. Excess incidence in the most recently published analysis from ERSPC remains higher than the CAP trial (crude cumulative incidence approximately 14% in the intervention group and 12% in the control group), but this is at least partly because the median follow‐up time after last screen in the analysis was only 8 (inter‐quartile range 5–12) years. For example, 8‐year follow‐up would only allow for up to half of screen‐detected cancers at a prevalence screen to have presented symptomatically, even if the man did not die over that period (Figure 1C). Therefore, the expected compensatory drop of cancer incidence in the screening arm of the ERSPC trial has not yet fully materialised in all participants. Indeed, a sub‐group analysis of ERSPC participants with the longest follow‐up since last screen (approximately 12 years) has similarly seen a continued narrowing of the excess incidence with follow‐up [12]. The importance of extended follow‐up has been seen in studies of overdiagnosis of lung and breast cancer [11, 20]. These analyses have shown that overdiagnosis estimates decrease with follow‐up time, and that variation in estimates of overdiagnosis between studies is largely explained by follow‐up time.

### 4.2 Limitations and Generalisability

There are some reasons our estimates may be too low for the target population. The most important of these is there was contamination in CAP from opportunistic PSA testing (as in the other trials) [1, 21, 22]. However, the trial was conducted and follow‐up data were collected in a period when opportunistic testing (which likely took place in both arms) was relatively low. The control group were also not directly informed about the trial, whereas the intervention group received some information about the trial and PSA testing. Therefore, we believe it is probable that the *differential* rate of asymptomatic testing subsequent to the screening is unlikely to change our main conclusions. Even if asymptomatic PSA testing only occurred in the control group at the maximum level estimated by the CAP triallists (15%), and no further PSA testing occurred at all to the initial 36% in the screening arm subsequently [13], overdiagnosis would not increase substantially if the estimate was adjusted upwards to reflect this. In an extreme situation where the net excess at 15 years is too low by a factor of (36 − 15)/36, then it would increase from 11.7% to 20.1%. Our estimates are also at risk of bias for repeat screening policies since they use data from a one‐off screening intervention. Cancers detected at subsequent screening rounds are expected to be, on average, identified earlier in the natural history of prostate cancer than those at a first round. In other words, the estimate is at risk of spectrum effects, such that one would expect the average lead time to be longer for cancer detected at subsequent screens. This would serve to slightly increase excess incidence relative to our analysis, and longer follow‐up would be needed than 15 years after last screen to reliably estimate overdiagnosis. Another potential issue is whether the results are generalisable to opportunistic testing in current practice. Opportunistic testing is screening but without organisation (including, e.g., invitations to the entire eligible population at regular intervals). While there may be some (self) selection effects from opportunistic populations that are hard to anticipate, we believe the findings are likely directly relevant.

There are some reasons our estimates may be too high. The first is that excess incidence might further decrease beyond 15 years (Figure 1D), and we have only considered follow‐up to 15 years after screening. Another is that the estimates effectively assume that no screen‐detected cancers would present with symptoms within 3 years of screening, since the net excess is 100% for the first 3 years. Therefore, there is a risk of bias in the denominator taken for proportion of screen detected cancers (it might be too small). It is also likely that men who received a PSA test in the intervention arm would be more likely to undergo subsequent PSA testing, or repeat (opportunistic) screening, which would serve to increase the excess between arms. The analysis also treats the uncensored lead‐time distribution to be the same by age, but other analyses have suggested that lead time may be less for younger men [1]. If true, overdiagnosis would be lower for younger men and even higher for older men. Lead time might also be shorter for men today because it is affected not only by age but also by biopsy protocols. Recent years have seen the development of further imaging and biomarker approaches that are used as secondary tests after a raised PSA to determine eligibility for biopsy. Randomised trials have shown that such approaches reduce detection of low‐grade cancers most likely to result in overdiagnosis [5, 23]. Another potential bias is from the relatively low uptake of screening in the intervention arm. This might make the estimate of overdiagnosis too high because attenders in CAP were more likely to live in areas of lower deprivation [24], and therefore to have previously had a PSA test and a higher risk of indolent disease than non‐attenders. Similarly, competing mortality and hence overdiagnosis risk will be slightly lower for the average man currently receiving PSA testing in the UK due to other demographic factors than age associated with socio‐economics, including comorbidities [25].

In summary, our re‐analysis of data from the CAP trial is at risk of bias in different directions, but taken together we suggest that the analysis of crude excess to 15 years and interpretation of the implications of this for overdiagnosis are likely to be reasonable. For example, the time of ~8 years to 50% excess, is consistent and no lower than other estimates of the average lead time from PSA testing in the pre‐MRI era [18]; and analysis of excess incidence in the Finnish component of ERSPC similarly found a continued decrease in excess incidence up to 12 years since screening, in the participants with the longest follow‐up since last (repeat) screen [12]. In addition, while the above considerations may affect the overall rate of overdiagnosis, they are less likely to affect the relative rates of overdiagnosis between different ages.

### 4.3 Policy Implications

There will be substantial overdiagnosis in men older than 70 years at screening, but much less overdiagnosis in younger men, for example, those aged 50–64 years. Screening older men is also unlikely to confer benefit in terms of mortality reduction. This was observed directly in the ERSPC trial where there was no benefit in the PSA arm for men aged 70–74 years (hazard ratio 1.06; 95% CI 0.80–1.41) [26]. This is because prostate cancer screening has a long lead time and there is relatively good survival from prostate cancer. Therefore, by the time a man could benefit from early screen detection older than 70 years, he is likely to have died from other causes—due to the high competing mortality rates in older men. We submit that it is unambigious that the trade‐off between benefits and harms from screening younger men is quite different than for older men.

The UK, like many other high‐income countries, has a policy that enables opportunistic screening. This is suboptimal because it has, effectively, led to screening in men older than 70 years [27, 28]. PSA testing rates in the UK in 2018 were three times higher for men aged 70–79 than 50–59 years [25, 29]. There is also a policy of PSA testing in older men with erectile dysfunction, haematuria and lower urinary tract symptoms (LUTS). The problem with this is that these are not symptoms of localised prostate cancer or associated with increased risk of high‐grade prostate cancer [30], and they are extremely common in older men [31]. Thus ‘symptomatic’ testing is effectively asymptomatic screening, also likely to result in overdiagnosis. The result of this and the opportunistic screening policy is that the number of older men receiving PSA tests has risen substantially in recent years [6, 32]. While there is some guidance on PSA testing for men aged 80 years or older [33], ensuring informed decision making is a challenge for primary care general practitioners. Men have access to conflicting information on harms and benefits such as from different websites, which is also reflected in differing interpretations of the evidence from various guideline developers [3, 4]. Some tools are available to support decision making focused on checking risk from prostate cancer; but none appear to evaluate risk of overdiagnosis based on a man's age. These could help ensure men in shared decision making contexts are adequately informed about risk of harm due to overdiagnosis from PSA testing in relation to their age and other life expectancy risk factors (see data availability section for an example app prototype).

Our analysis provides indirect support to the hypothesis that opportunistic testing, as currently implemented in the UK, is worse than a policy of either organised risk‐based screening [3], or no access to opportunistic PSA testing at all [34]. However, it is insufficient to determine what type of organised screening, if any, should be recommended in its place. A more comprehensive evaluation is needed for this, including to estimate the impact of substantially earlier diagnosis from screening younger men on their quality of life, and overall cost‐effectiveness. Other work has considered these issues, including to inform the recent UK NSC draft statement on prostate cancer screening [7]. The findings from our analysis provide a means to evaluate the face validity of overdiagnosis projections from this model. The model projected overdiagnosis from a one‐off screen to be 28% for a 50 years old, increasing to 82% for a 70 years old [7]; and an associated infographic gave a headline figure of 71% (20/28) for screening men aged 50–60 years [35]. Based on our analysis these are substantial overestimates, especially since MRI‐targeted biopsy was not part of the diagnostic pathway in CAP. Our empirical estimates of excess incidence from 15 years follow‐up match more closely other model‐based estimates of overdiagnosis [36]. We also note that the model used by UK NSC evaluated a policy of organised screening on top of a policy that enables opportunistic testing, which few would advocate irrespective of modelling. For example, the analysis did not consider that an organised programme could reduce overdiagnosis compared with opportunistic testing, by ending the availability of PSA testing through primary care for asymptomatic older men. Therefore, in our view, this model needs further work before it may be considered sufficiently reliable to project the impact of organised‐screening regimens on overdiagnosis relative to the current policy that enables opportunistic testing. Further data are also needed to evaluate the impact of harms from overdiagnosis in the modern era. This includes on the use of active surveillance [37], and focal therapy [38]. Ongoing trials are anticipated to provide such evidence to help further guide policymakers.

## Author Contributions

**Adam R. Brentnall:** conceptualization, methodology, data curation, software, formal analysis, writing – original draft, writing – review and editing. **Matejka Rebolj:** methodology, writing – review and editing. **Peter Sasieni:** methodology, writing – review and editing, funding acquisition. **Garth Funston:** methodology, writing – review and editing. **Rhian Gabe:** methodology, writing – review and editing. **Andrew J. Vickers:** conceptualization, methodology, writing – review and editing.

## Funding

The work of Dr. Andrew J. Vickers was supported in part by the National Institutes of Health/National Cancer Institute (NIH/NCI) with a Cancer Center Support Grant to Memorial Sloan Kettering Cancer Center (P30 CA008748), a SPORE grant in Prostate Cancer to Dr. H. Scher (P50‐CA92629). Matejka Rebolj acknowledges funding from Cancer Research UK (C8162/A29083 awarded to Peter Sasieni).

## Conflicts of Interest

A.R.B. declares grants outside this work from Prostate Cancer UK (including for the TRANSFORM trial of prostate cancer screening), NIHR, Breast Cancer Research Foundation, MRC, Cancer Research UK; Royalties from Cancer Research UK; Consulting fees from QM Innovation (Median Technologies) and Kings College London Talent Bank (NHS Galleri trial). M.R. declares grants outside this work from Cancer Research UK, Public Health England, and the UK National Screening Committee; fees for attendance and travel cost reimbursement at advisory board and other meetings organised by Hologic. P.S. declares grants outside this work from Yorkshire Cancer Research (IMProVE trial of prostate cancer screening), Cancer Research UK, NIHR. P.S. is a paid member of the scientific advisory board for GRAIL. G.F. reports research funding outside this work from NIHR, Cancer Research UK, and Prostate Cancer Research. R.G. reports grants outside this work from Prostate Cancer UK (including for the TRANSFORM trial of prostate cancer screening), NIHR, Yorkshire Cancer Research. A.J.V. is named on the patent for the statistical model that has been licensed and commercialised as the 4Kscore by OPKO Diagnostics, receives royalties from sales of this test, and owns stock options in OPKO.

## Data Availability

All data used and analysis code are available at https://github.com/brentnall/pca‐overdx, and an overdiagnosis calculator app prototype. Further information is available from the corresponding author upon request.
